# Insights Into Mechanisms of Biofilm Formation in *Acinetobacter baumannii* and Implications for Uropathogenesis

**DOI:** 10.3389/fcimb.2020.00253

**Published:** 2020-05-29

**Authors:** Jennifer M. Colquhoun, Philip N. Rather

**Affiliations:** ^1^Department of Microbiology and Immunology, Emory University, Atlanta, GA, United States; ^2^Research Service, Atlanta VA Healthcare System, Decatur, GA, United States

**Keywords:** *Acinetobacter baumannii*, bacterial biofilm, uropathogen, CAUTI, virulence, environmental sensing, gene expression

## Abstract

Multidrug resistant *Acinetobacter baumannii* is a serious healthcare threat. In fact, the Center for Disease Control recently reported that carbapenem-resistant *A. baumannii* is responsible for more than 8,500 infections, 700 deaths, and $281 million in healthcare costs annually in the United States with few, if any, treatment options available, leading to its designation as a pathogen of urgent concern and a priority for novel antimicrobial development. It is hypothesized that biofilms are, at least in part, responsible for the high prevalence of *A. baumannii* nosocomial and recurrent infections because they frequently contaminate hospital surfaces and patient indwelling devices; therefore, there has been a recent push for mechanistic understanding of biofilm formation, maturation and dispersal. However, most research has focused on *A. baumannii* pneumonia and bloodstream infections, despite a recent retrospective study showing that 17.1% of *A. baumannii* isolates compiled from clinical studies over the last two decades were obtained from urinary samples. This highlights that *A. baumannii* is an underappreciated uropathogen. The following minireview will examine our current understanding of *A. baumannii* biofilm formation and how this influences urinary tract colonization and pathogenesis.

## Introduction

*Acinetobacter baumannii* is a public health menace recently rising to prominence due to the rapid increase in antibiotic resistance and infection rates. Infections caused by *A. baumannii* account for ~2% of all healthcare-associated infections in the United States and Europe (Sievert et al., 2013; Magill et al., 2014; Lob et al., 2016) and this rate is nearly doubled in Asia and the Middle East (Lob et al., 2016). Globally, it is estimated that nearly 45% of all *A. baumannii* isolates are multidrug-resistant (MDR; resistant to ≥3 antibiotics) with rates as eclipsing 70% in Latin America and the Middle East (Giammanco et al., 2017). We have reached a critical tipping point where antibiotic discovery cannot keep up with the rapidly evolving antibiotic resistance of *A. baumannii* without some type of intervention. Hence, the World Health Organization (WHO) and Centers for Disease Control (CDC) have signified *A. baumannii* as a pathogen of critical importance for the discovery of novel antimicrobials (WHO, 2017; CDC, 2019).

*Acinetobacter baumannii* primarily causes infections of the lung or bloodstream (Peleg et al., 2008). However, it was recently reported that up to one-fifth of all *A. baumannii* isolates are obtained from urinary sources, implying that this organism is an underappreciated uropathogen (Di Venanzio et al., 2019). Catheter-acquired urinary tract infections (CAUTI) are one of the most common hospital-acquired infections accounting for an estimated 100,000 infections annually in the United States (Zarb et al., 2012; Magill et al., 2014). It is hypothesized that bacterial biofilm formation along the catheter surface is the most important factor in the establishment of bacteriuria (Stickler, 2008). *Acinetobacter baumannii*'s increasing prevalence in CAUTIs is due to its adept ability to form biofilms, with an estimated >75% of all isolates capable of forming a biofilm (Abdi-Ali et al., 2014; Azizi et al., 2016; Thummeepak et al., 2016). Therefore, understanding the mechanisms responsible for *A. baumannii* biofilm biogenesis and maturation are critical for elucidating the basis for uropathogenesis and may help with the development of future CAUTI anti-biofilm therapies. The following minireview examines existing data focused on the genetic regulation of *A. baumannii* biofilm lifestyle and its contribution to uropathogenesis as well as identifies current knowledge gaps to be addressed moving forward.

## Biofilm Formation

### Bacterial Cell Adherence

The initial step involved in the shift from planktonic to biofilm formation is surface contact and irreversible attachment (reviewed in Petrova and Sauer, 2012; Armbruster and Parsek, 2018). *Acinetobacter baumannii* has the ability to form biofilms on a wide range of surfaces including abiotic surfaces, like stainless steel and polypropylene, as well as host epithelial cells (Greene et al., 2016). Many virulence factors have been implicated in bacterial cell adherence, however the plasticity observed in *A. baumannii* genomes leads to significant strain-specific variations in biofilm formation. Investigation into the presence of known biofilm-associated genes in *A. baumannii* clinical isolates across several publications (Loehfelm et al., 2008; Badmasti et al., 2015; Zeighami et al., 2019) has shown that the most highly conserved genes were CsuE, the proposed tip subunit of the chaperone-usher pili (Csu), and OmpA (reported 81–100% detection). For the biofilm-associated protein (Bap) and class A extended β-lactamase blaPER-1 enzyme, detection was variable ranging from 30–66% to 2–64% of isolates, respectively. The Csu assembly system is composed of pilin subunits CsuA/B, CsuA, CsuB, and CsuE and transport proteins CsuC and CsuD, is highly conserved in biofilm-forming isolates and critical for adherence to abiotic surfaces, but not host surfaces (Tomaras et al., 2003; de Breij et al., 2009). Outer membrane protein A (OmpA) is a prominent porin that contributes to drug resistance, adhesion to epithelial cells and biofilm formation on plastic surfaces (C.H. Choi et al., 2008; Gaddy et al., 2009). Anti-OmpA serum and antibodies blocked *A. baumannii*'s adherence and subsequent invasion of host cells (Schweppe et al., 2015). Biofilm-associated protein (Bap) is a surface-exposed, highly divergent protein that is required for adherence to bronchial cells and structural integrity and water channel formation within the biofilm (Loehfelm et al., 2008; Brossard and Campagnari, 2012; De Gregorio et al., 2015). One study found that disruption of the Bap gene led to significant reductions in biofilm thickness and volume, interbacterial cell adhesion and ability to form higher order structures on medically relevant abiotic surfaces (Loehfelm et al., 2008). Another recent study found that the variation in the *bap* coding sequence across *A. baumannii* lineages results in differential functions during biofilm development with some versions displaying better adherence properties and others forming more complex biofilms (Skerniskyte et al., 2019). β-lactamase blaPER-1-expressing strains displayed significantly increased cell adhesiveness and biofilm formation compared to strains lacking the β-lactamase (H.W. Lee et al., 2008). However, additional publications report no or limited correlation between *blaPER-1* expression and biofilm formation (Sechi et al., 2004; Rao et al., 2008); thus, more research is required to elucidate its role.

Other virulence factors that have been implicated in adherence and biofilm formation include Pap, Prp, Cup, and Type IV pili systems as well as Acinetobacter trimeric autotransporter (Ata) (reviewed in Gaddy and Actis, 2009; Eijkelkamp et al., 2014; Longo et al., 2014; Harding et al., 2018). The *pap* operon encodes proteins homologous to P pili in *E. coli*, which has been found to be important for migration of bacteria from the bladder to the kidney (Wullt et al., 2000). The *prpABCD* operon encodes a photoregulated pilus associated with light-regulated motility and biofilm formation in ATCC 17978 (Wood et al., 2018). In addition, this operon is conserved in several other *A. baumannii* strains, including the hyper-biofilm forming MAR002, which displayed a 25-fold increase in the *prpD* homolog in sessile cells (Alvarez-Fraga et al., 2016). CUP2 pili were recently discovered as a *prp* operon homolog in UTI pathogen UPAB1, which when deleted resulted in reduced adhesion to both the catheter surface and bladder lumen in a CAUTI murine model (Di Venanzio et al., 2019). Type IV pili, encoded by the *pil* operon, have been shown to play a role in adhesion to cells and stainless steel (Ronish et al., 2019). Ata is a surface-exposed protein that has been shown to play an important role in biofilm formation as well as adherence to host cells and various host extracellular components (Bentancor et al., 2012; Weidensdorfer et al., 2019).

### Biofilm Formation Cues and Detection

Following adhesion to a surface, the bacterial cells are now primed to continue the shift to the biofilm state. The next step in biofilm formation involves environmental signal sensing and signal transduction, which will lead to downstream cellular responses. Many signals and signaling components that have been implicated in the control of biofilm formation and virulence factor production in *A. baumannii* are described below.

*Acinetobacter baumannii* and its close relative *Acinetobacter nosocomialis* have one quorum sensing (QS) system, which plays an integral role in regulating virulence factors, biofilm formation and surface motility (Niu et al., 2008; Clemmer et al., 2011; Bhargava et al., 2015; Subhadra et al., 2019). AbaI is the autoinducer synthase that generates the QS molecule N-(3-hydroxydodecanoyl)-L-HSL (AHL), which at high enough density interacts with the cognate receptor AbaR leading to downstream cellular responses. Several publications have found that AbaI and AbaR gene disruption leads to reduced biofilm formation (Niu et al., 2008; Anbazhagan et al., 2012; Guo and Xiang, 2017). Furthermore, cells cultured in the presence of AHL exhibited increased expression of Csu pili and stimulation of biofilm formation (Luo et al., 2015). Additionally, the activity of AbaI and biofilm production are regulated by iron in a dose-dependent manner (Modarresi et al., 2015), suggesting that iron is a possible environmental signal for nutrient limitation and the shift to survival mechanisms.

Several two component systems (TCS) have been shown to play a critical role in biofilm formation. BfmRS is predicted to contribute to the enhanced biofilm formation on abiotic surfaces since a knockout mutant of *bfmS* displayed drastic reduction in biofilm formation, adherence to eukaryotic cells and serum killing resistance compared to the wildtype strain (Liou et al., 2014). Furthermore, the *csu* operon is regulated by BfmRS, suggesting that the TCS plays an integral role in the initial adhesion step of biofilm formation (Tomaras et al., 2008; Shin et al., 2009). AdeRS is another TCS implicated in biofilm formation because an *adeS* deletion mutant resulted in decreased biofilm formation (Richmond et al., 2016). GacSA TCS was initially discovered for its role in citrate metabolism (Dorsey et al., 2002). However, further characterization of a *gacS* deletion mutant revealed its involvement in the control of pili synthesis, motility, biofilm formation, resistance against human serum, and metabolism of aromatic compounds by the *paa* operon (Cerqueira et al., 2014). Finally, A1S_2811 is a recently characterized hybrid sensor kinase expressed in an operon with *pilGHIJ* genes, suggesting a potential link to adhesion. Further, the *A1S_2811* deletion mutant displayed a significant reduction in surface motility, pellicle formation and *abaI* protein (Chen et al., 2017), suggesting a second putative control mechanism associated with QS.

Many other signals and sensing systems have been recently implicated in biofilm formation by *A. baumannii*. One study showed that cyclic di-GMP may play a role in *A. baumannii* biofilm formation since small molecule inhibitors of diguanylate cyclase enzymes (DGC) significantly reduced biofilm density (Sambanthamoorthy et al., 2014). Furthermore, another publication identified 2 DGCs that control biofilm and pellicle formation (Ahmad et al., 2020). When these enzymes are overexpressed, it drives early poly-N-acetyl-β-(1-6)-glucosamine (PNAG) production, which is an important biofilm extracellular matrix component. Temperature influences biofilm robustness since 26°C biofilms displayed significantly increased biofilm mass compared to 30 and 37°C (Eze and El Zowalaty, 2019). Mussi et al. showed that *A. baumannii* senses and responds to blue light as motility and biofilm formation were only observed in cultures grown in darkness, with the responsiveness level influenced by temperature (Mussi et al., 2010). The predicted photoreceptor protein is conserved in other *A. baumannii* strains, suggesting that light sensing is a potential widespread cue in *Acinetobacter* species. Deletion of A1S_0114 displayed an increase in *csuAB* expression as well as a decrease in other pilin proteins and *ompA* (Rumbo-Feal et al., 2017). Further, this mutant was unable to form complex 3D biofilm structures on abiotic surfaces and reduced airway epithelial adhesion. Recently, a Zur-regulated lipoprotein ZrlA was described to be involved in biofilm formation and motility through BfmRS signaling and subsequent control of *csu* expression (E.K. Lee et al., 2020).

### Transcriptomic and Proteomic Changes

**Figure 1:**
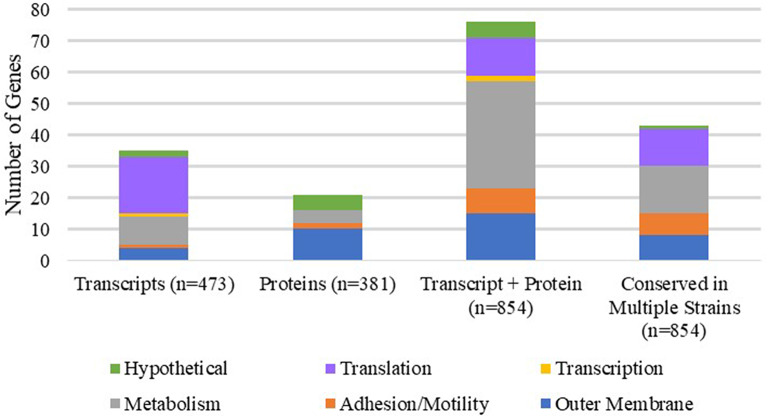
Functional categorization of corroborated genes up-regulated in biofilms. Each stacked bar represents the number of corroborated genes confirmed by transcriptional, proteomic, transcriptional and proteomic data sets (as listed in Table 1). The last stacked bar represents the number of genes confirmed in at least two different strains of *A. baumannii* regardless of data set. The total number of genes compared in each data set are listed after the bar title (i.e., 473 up-regulated transcripts compiled and compared, 381 up-regulated proteins compiled and compared, and so on). Each stacked bar is further broken down into functional subcategories: outer membrane proteins (blue), adhesion and motility (orange), metabolism (gray), transcription (yellow), translation (purple), and hypothetical proteins (green).

Several studies have compared the transcriptomic and proteomic profiles of *A. baumannii* grown in various growth conditions, including exponential, late stationary, pellicle and biofilm states, to elucidate the functional and metabolic differences between various bacterial lifestyles (Shin et al., 2009; Cabral et al., 2011; Marti et al., 2011; Chopra et al., 2013; Rumbo-Feal et al., 2013; Han et al., 2014; Kentache et al., 2017; Li et al., 2017; Penesyan et al., 2019). To gain more insight into the differential cellular response associated with biofilms, we compiled transcriptional and proteomic data reported from 9 publications, focusing specifically on up-regulated genes in biofilm/pellicle states compared to exponential growth (Table 1, Supplementary Tables 1, 2). Our efforts evaluated a total of 854 reported up-regulated genes (473 up-regulated transcripts and 381 up-regulated proteins) across 7 different *A. baumannii* strains (ATCC 17978, AB5075_UW, A077, A061, A132, 1656-2, BJAB0868), 3 of which were isolated from urinary sources (A077, A061, A132). Overall, we found 132 up-regulated genes to be corroborated between independent strains and/or separate publications (Table 1). Seventy-six genes were confirmed by both transcriptional and proteomic data with 43 of those genes validated across at least 2 different *A. baumannii* strains. Further, 35 and 21 genes were verified by at least two independent collections of transcriptional data and proteomic data, respectively. We further broke down these 132 up-regulated biofilm genes into basic biological function categories: Outer membrane proteins, Attachment/Motility, Metabolism, Transcription, Translation, and Hypothetical proteins (Table 1 and Figure 1). The largest represented categories were metabolism (49 genes), translation (30 genes), and outer membrane proteins (29 genes). This suggests that the transition and maintenance of the biofilm state involves significant changes to metabolic processes and outer membrane composition supported by translational machinery required to produce nascent proteins.

**Table 1:** Transcriptionally and/or proteomically corroborated genes up-regulated in biofilms.

			**Transcriptional**			**Proteomic**		
**Gene name**	**ATCC 17978 Gene ID**	**Gene description**	**Fold change biofilm vs. exponential phase cells^**A**^**	**Strains(s)^**B**^**	**References^**C**^**	Fold change biofilm/pellicle vs. exponential phase cells (^**^Unless noted PSM)^**D**^	**Strain(s)^**E**^**	**References^**F**^**
**TRANSCRIPT AND PROTEIN**								
**Outer membrane proteins**								
	A1S_0009	Putative RND type efflux pump	2.57	ATCC 17978	(Rumbo-Feal et al., 2013)	3.28, 3.23	ATCC 17978	Cabral et al., 2011; Kentache et al., 2017
	A1S_0116	RND superfamily exporter	56.18	ATCC 17978	(Rumbo-Feal et al., 2013)	3.49	ATCC 17978	Kentache et al., 2017
	A1S_0117	putative porin	23.97	ATCC 17978	(Rumbo-Feal et al., 2013)	7.95	ATCC 17978	Kentache et al., 2017
oprD	A1S_0201	outer membrane protein	3.08	AB5075_UW	(Penesyan et al., 2019)	3.3, 2.4	A077, ATCC 17978	Marti et al., 2011; Kentache et al., 2017
ompW	A1S_0292	outer membrane protein W	0.53	ATCC 17978	(Rumbo-Feal et al., 2013)	3.41, PSM 4, PSM 11	ATCC 17978, A077, A061	Nait Chabane et al., 2014; Kentache et al., 2017
gltP	A1S_0429	DAACS family glutamate:aspartate symporter	3.04	ATCC 17978	(Rumbo-Feal et al., 2013)	3.48	ATCC 17978	Kentache et al., 2017
fepA	A1S_0980	ferric enterobacter receptor	4.38	ATCC 17978	(Rumbo-Feal et al., 2013)	3.72, 2.99	ATCC 17978	Cabral et al., 2011; Kentache et al., 2017
putP	A1S_1530	SSS family major sodium/proline symporter	0.29	ATCC 17978	(Rumbo-Feal et al., 2013)	2.92	ATCC 17978	Kentache et al., 2017
bauB	A1S_2386	ferric acinetobactin binding protein	9.1	ATCC 17978	(Rumbo-Feal et al., 2013)	2.4, 2.5, 2.49	A077, ATCC 17978	Marti et al., 2011; Kentache et al., 2017
tolA	A1S_2591	tolerance to group A colicins single-stranded filamentous DNA phage	2.29	AB5075_UW	(Penesyan et al., 2019)	5.94	ATCC 17978	Kentache et al., 2017
ompA	A1S_2840	outer membrane protein A	0.6, 0.67	ATCC 17978, BJAB0868	(Rumbo-Feal et al., 2013; Li et al., 2017)	1.56, 2.11	ATCC 17978	Cabral et al., 2011
secY	A1S_3061	preprotein translocase	2.64, 4.35	AB5075_UW, ATCC 17978	(Rumbo-Feal et al., 2013; Penesyan et al., 2019)	2.47	ATCC 17978	Kentache et al., 2017
	A1S_3300	Na+/solute symporter	2.12, 17.44	AB5075_UW, ATCC 17978	(Rumbo-Feal et al., 2013; Penesyan et al., 2019)	4.68	ATCC 17978	Kentache et al., 2017
ddlB	A1S_3334	D-alanine/D-alanine ligase B	2.05	AB5075_UW	(Penesyan et al., 2019)	3.15	ATCC 17978	Kentache et al., 2017
bamC	A1S_3424	outer membrane assembly protein	2.18	AB5075_UW	(Penesyan et al., 2019)	3.16	ATCC 17978	Kentache et al., 2017
**Adhesion and motility**								
papC	A1S_1508	P pilus protein	29.85	BJAB0868	Li et al., 2017	1.6, 3.2	A077	(Marti et al., 2011)
papE	A1S_1510	fimbrial protein precursor	4.43, 4.12	MAR002	Alvarez-Fraga et al., 2016	PSM 35, PSM 67. PSM 28	A077, A061, A132	Nait Chabane et al., 2014
	A1S_2091	putative exported protein, FimA-like	24.78, 10, 24.98	ATCC 17978, MAR002, ATCC 17978	(Rumbo-Feal et al., 2013; Alvarez-Fraga et al., 2016)	PSM 90, PSM 101, PSM 37	A077, A061, A132	Nait Chabane et al., 2014
csuD	A1S_2214	chaperone usher pathway, type I pilus subunit	189.24, 180.04	BJAB0868, ATCC 17978	Rumbo-Feal et al., 2013; Li et al., 2017	3.53	ATCC 17978	(Kentache et al., 2017)
csuC	A1S_2215	chaperone usher pathway, type I pilus subunit	205.13, 201.23	BJAB0868, ATCC 17978	(Rumbo-Feal et al., 2013; Li et al., 2017)	2.8	A077	Marti et al., 2011
csuB	A1S_2216	chaperone usher pathway, type I pilus subunit	12.23, 11.96	BJAB0868, ATCC 17978	(Rumbo-Feal et al., 2013; Li et al., 2017)	PSM 25, PSM 32, PSM 22	A077, A061, A132	Nait Chabane et al., 2014
csuA	A1S_2217	chaperone usher pathway, type I pilus subunit	3.84	ATCC 17978	(Rumbo-Feal et al., 2013)	PSM 19, PSM 27, PSM 11	A077, A061, A132	Nait Chabane et al., 2014
csuA/B	A1S_2218	chaperone usher pathway, type I pilus subunit	0.34, 34.45, 164.4	MAR002, BJAB0868, ATCC 17978	(Rumbo-Feal et al., 2013; Alvarez-Fraga et al., 2016; Li et al., 2017)	25.28, PSM 406, PSM 429, PSM 399, 11.35	ATCC 17978, A077, A061, A132, ATCC 17978	(Cabral et al., 2011; Nait Chabane et al., 2014; Kentache et al., 2017)
**Metabolism**								
araT	A1S_0071	aromatic-amino-acid aminotransferase	2.31	AB5075_UW	(Penesyan et al., 2019)	2.06	ATCC 17978	Kentache et al., 2017
	A1S_0118	NAD-dependent epimerase/dehydratase; Carboxylesterase]	9.31	ATCC 17978	(Rumbo-Feal et al., 2013)	4.9	ATCC 17978	Kentache et al., 2017
atpF	A1S_0151	ATP synthase F0, B subunit	3.51, 1.9	AB5075_UW, ATCC 17978	Rumbo-Feal et al., 2013; Penesyan et al., 2019	PSM 32	A061	Nait Chabane et al., 2014
hom	A1S_0239	homoserine dehydrogenase	2.39	AB5075_UW	Penesyan et al., 2019	3.45	ATCC 17978	Kentache et al., 2017
ubiB	A1S_0348	2-octaprenylphenol hydroxylase of ubiquinone biosynthetic pathway	2.2	AB5075_UW	Penesyan et al., 2019	2.37	ATCC 17978	Kentache et al., 2017
nuoF	A1S_0756	NADH dehydrogenase I chain F	2.62	AB5075_UW	Penesyan et al., 2019	2.75	ATCC 17978	Kentache et al., 2017
hisZ	A1S_1178	ATP phosphoribosyltransferase	2.01	AB5075_UW	Penesyan et al., 2019	2.94	ATCC 17978	Kentache et al., 2017
	A1S_1267	lactam utilization protein	2.66	AB5075_UW	Penesyan et al., 2019	7.36	ATCC 17978	Kentache et al., 2017
	A1S_1269	allophanate hydrolase	3.78	AB5075_UW	Penesyan et al., 2019	6.55	ATCC 17978	Kentache et al., 2017
bccA	A1S_1270	carbamoyl-phosphate synthase	3.07	AB5075_UW	Penesyan et al., 2019	6.75	ATCC 17978	Kentache et al., 2017
paaZ	A1S_1335	aldehyde dehydrogenase	22.56	ATCC 17978	Rumbo-Feal et al., 2013	2.7	ATCC 17978	Kentache et al., 2017
paaA	A1S_1336	subunit A of Phenylacetate-CoA oxygenase	21.33	ATCC 17978	Rumbo-Feal et al., 2013	8.41	ATCC 17978	Kentache et al., 2017
paaB	A1S_1337	subunit B of Phenylacetate-CoA oxygenase	93.43	ATCC 17978	Rumbo-Feal et al., 2013	20.58	ATCC 17978	Kentache et al., 2017
paaC	A1S_1338	subunit C of Phenylacetate-CoA oxygenase	22.63	ATCC 17978	Rumbo-Feal et al., 2013	41.08	ATCC 17978	Kentache et al., 2017
paaE	A1S_1340	phenylacetate-CoA oxygenase/reductase subunit	34.73	ATCC 17978	Rumbo-Feal et al., 2013	PSM 22, 14.96	A132, ATCC 17978	Nait Chabane et al., 2014; Kentache et al., 2017
paaF	A1S_1341	enoyl-CoA hydratase/carnithine racemase	161.43	ATCC 17978	Rumbo-Feal et al., 2013	2.46	ATCC 17978	Kentache et al., 2017
paaJ	A1S_1344	beta-ketoadipyl CoA thiolase	28.43	ATCC 17978	Rumbo-Feal et al., 2013	16.96	ATCC 17978	Kentache et al., 2017
	A1S_1376	acyl-coA dehydrogenase	11.34	ATCC 17978	Rumbo-Feal et al., 2013	6.73	ATCC 17978	Kentache et al., 2017
acoA	A1S_1699	pyruvate/2-oxoglutarate dehydrogenase complex	3.28	ATCC 17978	Rumbo-Feal et al., 2013	10.77	ATCC 17978	Kentache et al., 2017
aspA	A1S_1726	aspartate ammonia-lyase	0.33	ATCC 17978	Rumbo-Feal et al., 2013	4.25	ATCC 17978	Kentache et al., 2017
atoD	A1S_1732	acetoacetyl-CoA transferase subunit alpha	78.74	ATCC 17978	Rumbo-Feal et al., 2013	2.63	ATCC 17978	Kentache et al., 2017
	A1S_2098	alcohol dehydrogenase	13.14	ATCC 17978	Rumbo-Feal et al., 2013	8.8	ATCC 17978	Kentache et al., 2017
ald1	A1S_2102	aldehyde dehydrogenase	2.59	ATCC 17978	Rumbo-Feal et al., 2013	17.72	ATCC 17978	Kentache et al., 2017
	A1S_2150	oxidoreductase	5.52	ATCC 17978	Rumbo-Feal et al., 2013	7.75	ATCC 17978	Kentache et al., 2017
cyoB	A1S_2167	cytochrome o ubiquinol oxidase subunit I	2.23	AB5075_UW	Penesyan et al., 2019	PSM 11, PSM 10, 2.22	A077, A061, ATCC 17978	Nait Chabane et al., 2014; Kentache et al., 2017
	A1S_2452	aldehyde dehydrogenase	1.71	ATCC 17978	Rumbo-Feal et al., 2013	4.94	ATCC 17978	Kentache et al., 2017
gltA	A1S_2710	citrate synthase I	3.33	ATCC 17978	Penesyan et al., 2019	PSM 33	A061	Nait Chabane et al., 2014
acs	A1S_3309	acetyl-coA synthetase	4.17	ATCC 17978	Rumbo-Feal et al., 2013	2.89	ATCC 17978	Kentache et al., 2017
xenB	A1S_3314	N-ethylmaleimide reductase	2.88	AB5075_UW	Penesyan et al., 2019	17.13	ATCC 17978	Kentache et al., 2017
hutG	A1S_3402	arginase/agmatinase/ formimionoglutamate hydrolase	2.03, 3.72	AB5075_UW, ATCC 17978	Rumbo-Feal et al., 2013; Penesyan et al., 2019	3.95	ATCC 17978	Kentache et al., 2017
hmgB	A1S_3413	fumarylacetoacetase	60.1	ATCC 17978	Rumbo-Feal et al., 2013	12.97	ATCC 17978	Kentache et al., 2017
hmgC	A1S_3415	maleylacetoacetate isomerase	24.49	ATCC 17978	Rumbo-Feal et al., 2013	19.1	ATCC 17978	Kentache et al., 2017
hmgA	A1S_3416	glyoxalase/bleomycin resistance protein/dioxygenas	24.26	ATCC 17978	Rumbo-Feal et al., 2013	6.58	ATCC 17978	Kentache et al., 2017
hpd	A1S_3418	4-hydroxyphenylpyruvate dioxygenase	78.62	ATCC 17978	Rumbo-Feal et al., 2013	16.83	ATCC 17978	Kentache et al., 2017
**Transcription**								
	A1S_2042	TetR family transcriptional regulator	2.72	ATCC 17978	Rumbo-Feal et al., 2013	4.99	ATCC 17978	Kentache et al., 2017
	A1S_2261	cold shock protein	5.09	ATCC 17978	Rumbo-Feal et al., 2013	2.22	ATCC 17978	Kentache et al., 2017
**Translation**								
thrC	A1S_0238	threonine synthase	2.15	AB5075_UW	Penesyan et al., 2019	2	ATCC 17978	Kentache et al., 2017
rplJ	A1S_0285	ribosomal protein L10	3.79, 2.31	AB5075_UW, ATCC 17978	Rumbo-Feal et al., 2013; Penesyan et al., 2019	PSM 54	A061	Nait Chabane et al., 2014
leuS	A1S_0541	leucyl-tRNA synthetase	2.02	AB5075_UW	Penesyan et al., 2019	2	ATCC 17978	Kentache et al., 2017
rplT	A1S_0597	50S ribosomal protein L20	4.14	AB5075_UW	Penesyan et al., 2019	PSM 12, PSM 54, PSM 17	A077, A061, A132	Nait Chabane et al., 2014
rpsG	A1S_0867	30S ribosomal protein S7	3.87	AB5075_UW	Penesyan et al., 2019	PSM 7, PSM 50, PSM 2	A077, A061, A132	Nait Chabane et al., 2014
tuf1	A1S_0869	elongation factor Tu	2.63, 1.22	AB5075_UW, ATCC 17978	Rumbo-Feal et al., 2013; Penesyan et al., 2019	PSM 11, PSM 45	A077, A061	Nait Chabane et al., 2014
rpsI	A1S_3001	30S ribosomal protein S9	3.93	AB5075_UW	Penesyan et al., 2019	PSM 39	A061	Nait Chabane et al., 2014
rpsD	A1S_3057	30S ribosomal protein S4	5.18, 1.89	AB5075_UW, ATCC 17978	Rumbo-Feal et al., 2013; Penesyan et al., 2019	PSM 36	A061	Nait Chabane et al., 2014
rplO	A1S_3062	50S ribosomal protein L15	4.17, 2.79	AB5075_UW, ATCC 17978	Rumbo-Feal et al., 2013; Penesyan et al., 2019	PSM 46	A061	Nait Chabane et al., 2014
rplE	A1S_3069	50S ribosomal protein L5	4.21, 2.13	AB5075_UW, ATCC 17978	Rumbo-Feal et al., 2013; Penesyan et al., 2019	PSM 33	A061	Nait Chabane et al., 2014
rplP	A1S_3074	50S ribosomal protein L16	4.92, 2.05	AB5075_UW, ATCC 17978	Rumbo-Feal et al., 2013; Penesyan et al., 2019	PSM 18	A061	Nait Chabane et al., 2014
rpsC	A1S_3075	30S ribosomal protein S3	5.45, 1.73	AB5075_UW, ATCC 17978	Rumbo-Feal et al., 2013; Penesyan et al., 2019	PSM 16, PSM 64, PSM 5	A077, A061, A132	Nait Chabane et al., 2014
**Hypothetical proteins**								
	A1S_1266	manganese transportor NRAMP	1.09	ATCC 17978	Rumbo-Feal et al., 2013	5.49	ATCC 17978	Kentache et al., 2017
	A1S_1268	hypothetical protein	2.9	AB5075_UW	Penesyan et al., 2019	5.34	ATCC 17978	Kentache et al., 2017
	A1S_1319	hypothetical protein	22.56	ATCC 17978	Rumbo-Feal et al., 2013	2.7	ATCC 17978	Kentache et al., 2017
	A1S_1932	hypothetical protein	1.88	ATCC 17978	Rumbo-Feal et al., 2013	3.74	ATCC 17978	Kentache et al., 2017
	A1S_2753	putative DcaP-like protein	1.66	ATCC 17978	Rumbo-Feal et al., 2013	2.01, 2.58, 1.70, 3.36	1656-2, ATCC 17978, A077, ATCC 17978	Shin et al., 2009; Cabral et al., 2011; Marti et al., 2011; Kentache et al., 2017
**TRANSCRIPT ONLY**								
**Outer membrane proteins**								
adeA	A1S_1751	multidrug efflux protein	4.05, 2.34	AB5075_UW, ATCC 17978	Rumbo-Feal et al., 2013; Penesyan et al., 2019			
adeT	A1S_1755	RND efflux pump subunit	17.27, 18.29	ATCC 17978, BJAB0868	Rumbo-Feal et al., 2013; Li et al., 2017			
basD	A1S_2382	ferric acquisition system	72.89, 79.98	ATCC 17978, BJAB0868	Rumbo-Feal et al., 2013; Li et al., 2017			
pstC	A1S_2447	phosphate ABC transporter	2.33, 7.56	AB5075_UW, ATCC 17978	Rumbo-Feal et al., 2013; Penesyan et al., 2019			
**Adhesion and motility**								
	A1S_1507	fimbrial protein	17.73, 1.95, 19	ATCC 17978, MAR002, MAR002	Rumbo-Feal et al., 2013; Alvarez-Fraga et al., 2016			
**Metabolism**								
prpB	A1S_0073	2-methylisocitrate lyase	3.29, 6.6	AB5075_UW, ATCC 17978	Rumbo-Feal et al., 2013; Penesyan et al., 2019			
atpA	A1S_0153	ATP synthase F1, alpha subunit	3.39, 1.13	AB5075_UW, ATCC 17978	Rumbo-Feal et al., 2013; Penesyan et al., 2019			
atpG	A1S_0154	ATP synthase F1, gamma subunit	3.26, 1.65	AB5075_UW, ATCC 17978	Rumbo-Feal et al., 2013; Penesyan et al., 2019			
atpD	A1S_0155	ATP synthase F1, beta subunit	3.49, 1.06	AB5075_UW, ATCC 17978	Rumbo-Feal et al., 2013; Penesyan et al., 2019			
atpC	A1S_0156	ATP synthase F1, epsilon subunit	3.77, 1	AB5075_UW, ATCC 17978	Rumbo-Feal et al., 2013; Penesyan et al., 2019			
pta	A1S_0481	phosphate acetyltransferase	2.36, 3.92	AB5075_UW, ATCC 17978	Rumbo-Feal et al., 2013; Penesyan et al., 2019			
	A1S_3231	acetyl-CoA hydrolase/transferase	2.28, 3.42	AB5075_UW, ATCC 17978	Rumbo-Feal et al., 2013; Penesyan et al., 2019			
hutH	A1S_3405	histidine ammonia-lyase	2.03, 3.72	AB5075_UW, ATCC 17978	Rumbo-Feal et al., 2013; Penesyan et al., 2019			
hutU	A1S_3406	urocanate hydratase	2.56, 3.92	AB5075_UW, ATCC 17978	Rumbo-Feal et al., 2013; Penesyan et al., 2019			
**Transcription**								
	A1S_3104	DEAD/DEAH box helicase	6.00, 1.64	AB5075_UW, ATCC 17978	Rumbo-Feal et al., 2013; Penesyan et al., 2019			
**Translation**								
tuf2	A1S_0279	elongation factor Tu	2.43, 1.18	AB5075_UW, ATCC 17978	Rumbo-Feal et al., 2013; Penesyan et al., 2019			
rplK	A1S_0283	50S ribosomal protein L11	4.11, 1.99	AB5075_UW, ATCC 17978	Rumbo-Feal et al., 2013; Penesyan et al., 2019			
rpsA	A1S_1572	30S ribosomal protein S1	2.00, 17.74	AB5075_UW, ATCC 17978	Rumbo-Feal et al., 2013; Penesyan et al., 2019			
tsf	A1S_2322	elongation factor Ts	2.54, 1.5	AB5075_UW, ATCC 17978	Rumbo-Feal et al., 2013; Penesyan et al., 2019			
rplQ	A1S_3055	50S ribosomal protein L17	5.49, 2.54	AB5075_UW, ATCC 17978	Rumbo-Feal et al., 2013; Penesyan et al., 2019			
rpoA	A1S_3056	DNA-directed RNA polymerase, alpha subunit	5.36, 1.89	AB5075_UW, ATCC 17978	Rumbo-Feal et al., 2013; Penesyan et al., 2019			
rpsK	A1S_3058	30S ribosomal protein S11	5.13, 2.00	AB5075_UW, ATCC 17978	Rumbo-Feal et al., 2013; Penesyan et al., 2019			
rpmD	A1S_3063	50S ribosomal protein L30	4.40, 2.54	AB5075_UW, ATCC 17978	Rumbo-Feal et al., 2013; Penesyan et al., 2019			
rpsE	A1S_3064	30S ribosomal protein S5	4.54, 2.97	AB5075_UW, ATCC 17978	Rumbo-Feal et al., 2013; Penesyan et al., 2019			
rplR	A1S_3065	50S ribosomal protein L18	4.71, 3.37	AB5075_UW, ATCC 17978	Rumbo-Feal et al., 2013; Penesyan et al., 2019			
rplF	A1S_3066	50S ribosomal protein L6	4.53, 2.55	AB5075_UW, ATCC 17978	Rumbo-Feal et al., 2013; Penesyan et al., 2019			
rpsN	A1S_3068	30S ribosomal protein S14	4.53, 2.55	AB5075_UW, ATCC 17978	Rumbo-Feal et al., 2013; Penesyan et al., 2019			
rplX	A1S_3070	50S ribosomal protein L24	3.78, 2.26	AB5075_UW, ATCC 17978	Rumbo-Feal et al., 2013; Penesyan et al., 2019			
rpmC	A1S_3073	50S ribosomal protein L29	4.45, 2.01	AB5075_UW, ATCC 17978	Rumbo-Feal et al., 2013; Penesyan et al., 2019			
rplB	A1S_3077	50S ribosomal protein L2	5.63, 1.75	AB5075_UW, ATCC 17978	Rumbo-Feal et al., 2013; Penesyan et al., 2019			
rplD	A1S_3079	50S ribosomal protein L4	5.76, 1.87	AB5075_UW, ATCC 17978	Rumbo-Feal et al., 2013; Penesyan et al., 2019			
rplC	A1S_3080	50S ribosomal protein L3	5.06, 2.13	AB5075_UW, ATCC 17978	Rumbo-Feal et al., 2013; Penesyan et al., 2019			
rplS	A1S_3161	50S ribosomal protein L19	3.95, 2.57	AB5075_UW, ATCC 17978	Rumbo-Feal et al., 2013; Penesyan et al., 2019			
**Hypothetical proteins**								
	A1S_0032	putative signal peptide	3.71, 32.18	AB5075_UW, ATCC 17978	Penesyan et al., 2019, Rumbo-Feal et al., 2013			
	A1S_2889	putative signal peptide	4.14, 46.5	AB5075_UW, ATCC 17978	Penesyan et al., 2019, Rumbo-Feal et al., 2013			
**PROTEIN ONLY**								
**Outer membrane proteins**								
oprC	A1S_0170	outer membrane copper receptor				7.47, 2.90, 5.86	1656-2, A077, ATCC 17978	Shin et al., 2009; Marti et al., 2011; Kentache et al., 2017
	A1S_0474	ferric siderophore receptor protein				1.80, 2.80, 3.17	A077, A077, ATCC 17978	Marti et al., 2011; Kentache et al., 2017
lysM	A1S_0820	peptidoglycan-binding LysM				PSM 19, PSM 47, PSM 10, 2.03	A077, A061, A132, ATCC 17978	Nait Chabane et al., 2014; Kentache et al., 2017
lolB	A1S_0835	LolB outer membrane lipoprotein precursor				PSM 9, PSM 9, PSM 7	A077, A061, A132	Nait Chabane et al., 2014
pfeA	A1S_0981	ferric enterobactin receptor precursor (part 2)				2.00, 2.84	A077, ATCC 17978	Marti et al., 2011; Kentache et al., 2017
	A1S_1063	TonB-dependent siderophore receptor precursor				1.90, 7.72	A077, ATCC 17978	Marti et al., 2011; Kentache et al., 2017
	A1S_1655	ferric siderophore receptor protein				3.50, 4.47	A077, ATCC 17978	Marti et al., 2011; Kentache et al., 2017
carO	A1S_2538	carbapenem-associated resistance protein precursor				3.03, 3.84, 4.12, 2.00, 2.80	ATCC 17978, A077	Cabral et al., 2011; Marti et al., 2011
	A1S_2773	putative long-chain fatty acid transport protein				3.70, PSM 15, PSM 26, PSM 19, 3.62	A077, A077, A061, A132, ATCC 17978	Marti et al., 2011; Nait Chabane et al., 2014; Kentache et al., 2017
mscL	A1S_2834	mechanosensitive channel				PSM 26, PSM 5, 2.05	A061, A132, ATCC 17978	Nait Chabane et al., 2014; Kentache et al., 2017
**Adhesion and motility**								
filF	A1S_0695	pilus assembly protein				2.20, 2.60, 2.70, 3.10, 2.78	A077, A077, A077, A077, ATCC 17978	Marti et al., 2011; Kentache et al., 2017
csuE	A1S_2213	chaperone usher pathway, type I pilus subunit				PSM 26, PSM 34, PSM 23	A077, A061, A132	Nait Chabane et al., 2014
**Metabolism**								
	A1S_1951	quinoprotein glucose dehydrogenase				3.48, 1.70	ATCC 17978, A077	Cabral et al., 2011; Marti et al., 2011
	A1S_1966	(3R)-hydroxymyristoyl-[acyl carrier protein] dehydratase				PSM 23, PSM 16	A077, A061	Nait Chabane et al., 2014
sdhC	A1S_2711	succinate dehydrogenase, cytochrome b556 subunit				PSM 14, PSM 29, PSM 10	A077, A061, A132	Nait Chabane et al., 2014
hisA	A1S_3238	acetyl-CoA hydrolase/transferase				PSM found on in biofilm sample, 4.06	1656-2, ATCC 17978	Shin et al., 2009; Kentache et al., 2017
**Hypothetical proteins**								
	A1S_0779	conserved hypothetical protein				PSM 39, PSM 68, PSM 30	A077, A061, A132	Nait Chabane et al., 2014
	A1S_1183	conserved hypothetical protein				PSM 39, PSM 47, PSM 28	A077, A061, A132	Nait Chabane et al., 2014
	A1S_2491	putative signal peptide				2.37, 4.94	1656-2, ATCC 17978	Shin et al., 2009; Kentache et al., 2017
	A1S_3343	conserved hypothetical protein, putative exported protein				PSM 8, PSM 8	A077, A061	Nait Chabane et al., 2014
	A1S_3384	conserved hypothetical protein				PSM 23, PSM 52, PSM 45	A077, A061, A132	Nait Chabane et al., 2014

*ATCC 17978 gene name, gene IDs, and gene description are listed first by combination of confirmed data with the first section presenting genes confirmed by both transcriptional and proteomic data sets, the second section confirmed only transcriptionally and the third section confirmed only proteomically. The up-regulated genes are then broken down into functional subcategories: outer membrane proteins, adhesion/motility, metabolism, transcription, translation, and hypothetical proteins (visual representation of Table 1 is presented in Figure 1). In the transcriptional data column, we compiled (A) fold change of the transcript in biofilm cells compared to exponential phase control cells, (B) the A. baumannii strain tested and (C) the publication from with the data was obtained. In the proteomic data column, we compiled (D) fold change or protein spectra match (PSM) of the protein in biofilm/pellicle cells compared to exponential phase control cells, (E) the A. baumannii strain tested and (F) the publication from with the data was obtained. If there is more than one publication reporting up-regulation of the same gene, the fold-changes, strains, and publications are separated by commas (,) unless the same strain was used across multiple publications. The first listed fold-change number reported corresponds to the first listed strain and publication and so on. All compiled data, including more information on the original gene ID call and biofilm/pellicle growth conditions, can be found in Supplementary Tables 1, 2*.

** *We were not able to integrate all the transcriptional changes observed in Li et al. (2017) since the reported gene code did not align to any sequenced A. baumannii genome. We selected genes that had clear gene annotations relevant to the compiled data set*.

The most highly upregulated genes observed in biofilm associated cells were the *csu* operon (*csuABCDE*) exhibiting overexpression levels ranging from 11- to 205-fold increase over exponential phase cells. The other attachment/motility genes identified to be upregulated include pili genes *filF, fimA*, and *papCE*. In the metabolic category, we observed the significant upregulation of the phenylacetate degradation operon (*paaZABCEFJ*), which has been linked to neutrophil evasion and regulation by the GacS/GacA TCS (Cerqueira et al., 2014). Of the 30 translational genes up-regulated, 24 are components composing the small and large subunits of the ribosome, suggesting an overall increase in translational capacity within biofilm cells. In the outer membrane protein category, we observe significant increases in RND efflux pump proteins and iron acquisition systems, which are important for intrinsic antibiotic resistance and nutrient procurement.

Though not confirmed in our gene list, the *pgaABCD* operon encodes the enzymes that produce PNAG, an important structural component for biofilm formation (A.H. Choi et al., 2009). Further, it has been shown that expression of *pgaB* is positively correlated with biofilm formation capacity in clinical isolates from burn wound infections in Iran (Amin et al., 2019). It is clear that the *A. baumannii* growth state results in different transcriptional, proteomic, and metabolic profiles, which account for variable cellular responses.

### Recent Developments

As mentioned previously, *A. baumannii* has only just begun to be recognized as an important uropathogen. A recent study discovered that a large conjugative plasmid (pAB5) in the MDR *A. baumannii* urinary isolate UPAB1 increases virulence in a first-of-its-kind CAUTI murine model of infection (Di Venanzio et al., 2019). Furthermore, UPAB1 grew better than ATCC 19606 in pooled human urine *in vitro* and co-localized with fibrinogen similar to previous observations in common UTI pathogens such as *E. faecalis* and MRSA (Walker et al., 2017; Xu et al., 2017). To identify adhesins involved in colonization of the bladder, Di Venanzio et al. identified two loci encoding putative CUP pili (CUP1 and CUP2). Deletion of these operons revealed loss of distinct surface appendages observed in the wildtype control and reduction in bacterial burden both on the catheter implant and within the bladder. Further, loss of pAB5 resulted in significantly reduced bacterial burden on the implant and within the bladder; however, the presence of pAB5 attenuated virulence and dissemination to other organs in an acute pneumonia murine model, which led the researchers to conclude that pAB5 confers niche specificity. To identify potential virulence factors differentially regulated by pAB5, researchers utilized proteomic and transcriptional approaches. Overall, their data indicated that pAB5 repressed type VI secretion system and differential regulation of PNAG biosynthesis and CUP1/2 pili are influenced by growth condition; thus, indicating that plasmid-encoded genes may influence biofilm formation and uropathogenesis by modulating the expression of chromosomal genes.

Another recent publication supports the hypothesis of niche-specific plasmid acquisition. They found distinct genome expansions in strains isolated from the similar sites of infections whereas strains isolated from another site of infection maintained different plasmids (Yakkala et al., 2019).

Given the wide-ranging phenotypic changes observed during the transition from planktonic to biofilm growth, it is likely that there are many levels of regulation involved in coordinating the cellular response. In recent years, the role of small RNAs (sRNA) in transcriptional regulation networks have been increasingly recognized. To this end, Alvarez-Fraga et al. compared the expression of sRNAs in ATCC 17978 biofilm cells and found 60 sRNAs were differentially regulated compared to planktonic cells (Alvarez-Fraga et al., 2017). Additionally, they were able to show that sRNA 13573 is involved in the biofilm formation and attachment to eukaryotic cells, suggesting that biofilm biogenesis and adhesion properties in ATCC 17978 are coordinately regulated. Interestingly, another group found a distinct set of differentially expressed sRNAs in *A. baumannii* strain MTCC1425 compared to ATCC 17978, suggesting that the sRNAs involved in transcriptional control display some strain specificity (Sharma et al., 2014).

Mangas et al. compared nearly 2000 *A. baumannii* genomes. They observed that strains carrying CRISPR systems were enriched for biofilm-associated genes (>70 vs. <2% non-CRISPR strains), suggesting a link between CRISPR immunity and biofilm formation (Mangas et al., 2019). Previous research has shown that Cas3 endonuclease is involved in the control of biofilm formation in both gram-positive and gram-negative bacteria (Tang et al., 2019; Cui et al., 2020).

## Perspectives

While investigations into the mechanisms behind *A. baumannii* biofilm formation and CAUTI-associated pathogenesis have expanded recently, there remains many questions left to be addressed in order to produce a fully developed model.

A general concern across all pathogenic organism studies is that *in vitro* assays have been important for identifying virulence factors responsible for pathogenesis. However, studies within animal models of these putative virulence factors have often lacked direct correlation with *in vivo* outcomes, including in *A. baumannii* studies (Wand et al., 2012; Giannouli et al., 2013; Zimbler et al., 2013; Beceiro et al., 2014; Lazaro-Diez et al., 2016). These results highlight the importance of the confirmation of virulence *in vivo*, especially in models reflecting human infection. The first CAUTI murine infection model was recently established and requires more investigation for validation (Di Venanzio et al., 2019), but is a good first step in addressing this concern.

Another major complication that is evident across the array of *A. baumannii* pathogenesis publications is that some of the biological roles associated with identified virulence factors seem to be strain specific. For example, Wood et al. described and characterized a light-regulated pilus system involved in ATCC 17978 biofilm formation; however, this operon displayed no changes in expression in the hyper-biofilm producing strain MAR002 (Alvarez-Fraga et al., 2016; Wood et al., 2018). Further, Eze and El Zowalaty observed significant strain variation in biofilm formation across strains tested under differing temperatures, nutrient levels and agitation conditions (Eze and El Zowalaty, 2019). Future work should investigate conservation and incorporate several different *A. baumannii* lineages to strengthen the original discovery.

One observation we encountered while compiling up-regulated genes involved in biofilm cell growth is the wide variation in methods used to measure biofilm formation (Shin et al., 2009; Cabral et al., 2011; Marti et al., 2011; Rumbo-Feal et al., 2013; Nait Chabane et al., 2014; Alvarez-Fraga et al., 2016; Kentache et al., 2017; Li et al., 2017; Penesyan et al., 2019). Publications reported using different incubation times (24–144 h), incubation temperatures (25–37°C), abiotic surfaces supplied (glass, polystyrene), and growth conditions (continuous flow, stationary). While we were able to identify a large set of genes up-regulated in biofilm cells despite differential growth conditions, we are concerned that many other genes may have been missed in these studies. For example, previously reported biofilm-associated genes, *bap* and the *pga* operon, were not reported to be up-regulated in any publication examined. Moving forward, transcriptional and proteomic profiling over time during biofilm formation and maturation will provide important information into the dynamic, rapidly transitioning cellular responses within sessile cells.

Recently, a novel, phase-variable colony opacity switch has been described in AB5075 and other *A. baumannii* clinical isolates, in which colonies interconvert at a high-frequency between opaque and translucent variants (Tipton et al., 2015). Further characterization of the two opacity forms showed significant differences in biofilm formation, virulence and transcriptional profiles (Chin et al., 2018). However, none of the publications discussed in this review mentioned focusing a specific phase variant, which likely means their results were generated from a mixed pool of cell types. This implies that transcriptional and proteomic data may be missing important differences since the average of the mixed population may match control even though one subpopulation could have the gene significantly up-regulated and the other subpopulation has the gene significantly down. This leads us to wonder what the individual contributions that each of the phase types have in biofilm formation and maturation. It is important to note that we have not observed colony opacity variation in ATCC 17978 and therefore, this phenotypic variation may not apply to studies using this strain.

Finally, there are nearly 2500 *A. baumannii* genome sequences publicly available comprising a core genome of ~2,200 genes and a collapsed pan-genome size of almost 20,000 genes (Chan et al., 2015; Mangas et al., 2019), showing the broad variation across this pathogen. Furthermore, 42% of the pan-genome is of unknown function displaying our superficial knowledge of the roles these genes play in *A. baumannii* growth, virulence and environmental adaptability. Overall, the accumulation of decades of research has revealed many genes that are involved in the transition from planktonic growth to biofilms in *A. baumannii*. Only recently has this organism begun to be appreciated as a uropathogen and research into this area has commenced. Many more studies are required to fully understand how biofilm-associated genes may contribute to urinary tract infection. As we gain more insight into the underlying mechanisms of biofilm formation and uropathogenesis, this work will lay the foundation for potential anti-infective targets to combat surmounting obstacle of MDR *A. baumannii*.

## Author Contributions

JC conceived and wrote the majority of the manuscript. PR contributed to manuscript revision. Both authors read and approved the submitted version.

## Conflict of Interest

The authors declare that the research was conducted in the absence of any commercial or financial relationships that could be construed as a potential conflict of interest.
